# Skeletal muscle aging: influence of oxidative stress and physical exercise

**DOI:** 10.18632/oncotarget.14670

**Published:** 2017-01-15

**Authors:** Mariana Janini Gomes, Paula Felippe Martinez, Luana Urbano Pagan, Ricardo Luiz Damatto, Marcelo Diacardia Mariano Cezar, Aline Regina Ruiz Lima, Katashi Okoshi, Marina Politi Okoshi

**Affiliations:** ^1^ Botucatu Medical School, Internal Medicine Departament, Sao Paulo State University, UNESP, Botucatu, SP, Brazil; ^2^ School of Physical Therapy, Federal University of Mato Grosso do Sul, Campo Grande, Brazil

**Keywords:** elderly, physical capacity, sarcopenia, training, treatment

## Abstract

Skeletal muscle abnormalities are responsible for significant disability in the elderly. Sarcopenia is the main alteration occurring during senescence and a key public health issue as it predicts frailty, poor quality of life, and mortality. Several factors such as reduced physical activity, hormonal changes, insulin resistance, genetic susceptibility, appetite loss, and nutritional deficiencies are involved in the physiopathology of muscle changes. Sarcopenia is characterized by structural, biochemical, molecular and functional muscle changes. An imbalance between anabolic and catabolic intracellular signaling pathways and an increase in oxidative stress both play important roles in muscle abnormalities. Currently, despite the discovery of new targets and development of new drugs, nonpharmacological therapies such as physical exercise and nutritional support are considered the basis for prevention and treatment of age-associated muscle abnormalities. There has been an increase in information on signaling pathways beneficially modulated by exercise; nonetheless, studies are needed to establish the best type, intensity, and frequency of exercise to prevent or treat age-induced skeletal muscle alterations.

## INTRODUCTION

With improved life conditions in the population and the availability of treatments for various diseases, particularly infectious and cardiovascular diseases, life expectancy and consequently the number of elderly in the population has increased [1]. Worldwide the 60 years and over population is predicted to rise from 841 million in 2013 to more than 2 billion by 2050 [2]. Muscle tissue has a wide range of functions and skeletal muscle abnormalities are responsible for significant disability in the elderly [3]. Sarcopenia is an important alteration occurring during senescence and a key public health issue as it predicts frailty, poor quality of life, and mortality [4–7]. The prevalence of low muscle mass is estimated to be between 8 and 40% depending on the population studied and the methods used to identify sarcopenia [8]. It ranges from 15% at 65 years to 50% at 80 years [6, 7, 9]. Although several factors can be involved, the physiopathology of muscle changes in healthy aging is not completely understood. Disruption of signaling networks involving systemic and skeletal muscle reactive oxygen species (ROS) has received increasing attention in recent decades [10]. Physical exercise is widely considered an important intervention for increasing longevity and promoting well-being and healthy aging.

In this review we first present the definition of sarcopenia. We then discuss the pathophysiological and molecular mechanisms underlying muscle changes during aging highlighting the role of oxidative stress. And finally, as strategies to prevent and treat age-associated muscle changes, we emphasize the role of physical exercise and introduce newer agents being developed.

## DEFINITION OF SARCOPENIA

Sarcopenia is characterized by age-associated progressive and generalized skeletal muscle mass and function loss occurring in the absence of underlying diseases. The definition of sarcopenia is still a matter of controversy and an evolving concept. In accordance with the European Working Group on Sarcopenia in Older People, sarcopenia diagnosis requires documentation of both low muscle mass and low muscle function (strength or performance) [11]. Low muscle mass was established as lean appendicular mass corrected for height squared of 2 or more standard deviations below the mean for healthy persons between 20 and 30 years of age from the same ethnic group [12, 13]. By using this definition diagnosis depends on laboratory investigation. Although dual-energy X-ray absorptiometry and bioelectrical impedance have been used to evaluate body composition and estimate total, lean and fat mass, computed tomography and magnetic resonance imaging are the gold standard for muscle mass assessment [14, 15]. As these techniques are expensive, the approach recommended by the International Working Group on Sarcopenia and the Foundation for the National Institutes of Health Sarcopenia Project requires the presence of either low physical performance or muscle strength as indications to measure muscle mass [9, 16].

For clinical purposes, ranges of cut-points were proposed to evaluate physical performance, muscle strength, and appendicular lean mass. A gait speed lower than 0.8 m/s to walk a set distance, such as 4 m, at usual pace has been used to determine low physical performance [11, 12]. Muscle strength can be evaluated by several different ways [11]. Grip strength is the most practical method in clinical settings and it correlates with lower limb physical performance measurements [17]. Grip strength cut-points have been proposed; they are 26-30 kg in men and 16-19 kg in women [3, 18]. Interestingly, observations have shown that strength may predict the risk of disability and mortality better than muscle mass [19, 20]. Cut-points for skeletal muscle mass vary according to population, medical societies and the methods used to normalize muscle mass, whether height squared or body mass index [18]. An appendicular skeletal muscle mass (kg) divided by height (m) squared below 7.26 kg/m^2^ in men and 5.45 kg/m^2^ in women has been used to diagnose sarcopenia [16, 21].

Sarcopenia should be differentiated from muscle loss associated with chronic disease, which is preferably called muscle wasting. Distinct syndromes with prominent muscle wasting include cachexia, frailty, and sarcopenic obesity [14, 22]. Cachexia is characterized by body weight, fat, and muscle loss due to an underlying illness [4]. Frailty, also associated with medical comorbidities, has been empirically characterized by weight loss, slowness, exhaustion, low physical activity, and weakness. Three of these frailty indicators are required to define the full frailty syndrome [23]. Sarcopenic obesity is the co-existence of obesity and sarcopenia. Lipid infiltration in muscle tissues exacerbates sarcopenia by preventing amino acids incorporation and protein synthesis [24, 25].

## AGING-ASSOCIATED SKELETAL MUSCLE ALTERATIONS

The main alteration associated with aging is muscle atrophy. Progressive muscle mass loss starts at approximately the age of 40 years; it is estimated at about 8% per decade until the age of 70 years and then it increases to 15% per decade [6]. Reduction in muscle mass is combined with an increase in body fat mass; consequently, body weight usually remains unchanged.

Several underlying structural and biochemical changes in muscle have been described in the elderly. Muscle cross-sectional area can be up to 30% less at 70 years than at 20 years old and is associated with an accumulation of fat within muscle [26, 27]. A shift in muscle fiber composition occurs in advancing age with a decrease in large fast-twitch glycolytic (Type II) fiber [9]. Changes in motor neurons have also been observed; with ageing, the number and activity of motor units are decreased impairing motor control [28, 29]. Alterations in the type of fibers may occur when type II myofibers are re-innervated by type I motor neurons [28]. However, whether this motor unit change is a cause of sarcopenia or a compensatory adaptive response to sarcopenia is unresolved [12].

As a consequence of structural and biochemical changes, muscle strength and functional capacity are reduced in the sarcopenic elderly [30]. The Health and Body Composition Study including 1880 older subjects showed a strong association between muscle mass and strength [31]. Leg strength decreases 10-15% per decade until 70 years of age, and then it declines 25% to 40% by decade [6]. Muscle strength is approximately 20-40% lower at 70 years than in young adults [27]. Reduction in muscle function is an important issue in clinical settings as it is independently associated with increased risk of falls, disability, and mortality in the elderly [32].

## ETIOLOGY AND INTRACELLULAR PATHWAYS INVOLVED IN AGING-ASSOCIATED MUSCLE CHANGES

Muscle loss is a multifactorial and not completely understood condition which occurs in the elderly and several systemic diseases [33, 35]. Although its main causative agents include reduced physical activity [36, 37], hormonal changes [38], insulin resistance [39, 40], genetic susceptibility [41], appetite loss and nutritional deficiencies [42, 43], their contribution to the normal aging process has not been fully determined.

**Figure 1:**
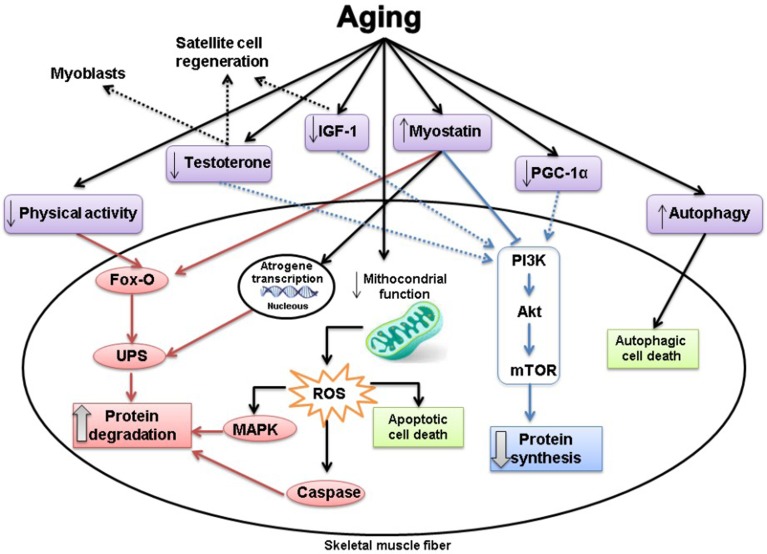
The effects of aging on the signalling pathways associated with protein synthesis and protein degradation Red: catabolic pathways. Blue: anabolic pathways. Dash lines: inhibition. Dotted lines: no stimulation. The main alteration associated with aging is muscle atrophy. Muscle loss results from a disproportionate decrease in muscle protein synthesis and/or an increase in protein breakdown. Protein synthesis and degradation are regulated by several different stimuli, which activate multiple signaling pathways. See the main text for further details.

Physiological maintenance of skeletal muscle mass depends on a delicate balance between anabolic and catabolic factors. Muscle loss results from a disproportionate decrease in muscle protein synthesis and/or an increase in protein breakdown (Figure 1) [9]. There is substantial evidence that anabolic drive is reduced in ageing [12]. An important anabolic pathway inducing protein synthesis involves activation of the phosphatidylinositol 3-kinase (PI3K)/serine threonine kinase (Akt), which stimulates mammalian target of rapamycin (mTOR) [44]. Most anabolic stimuli, such as: insulin and insulin-like growth factor 1 (IGF-1), exercise, and testosterone, upregulate this pathway [45]. As aging is associated with a sedentary lifestyle, IGF-1 and insulin resistance, and lower testosterone levels, this pathway is inhibited and muscle protein synthesis is blunted [4]. A vicious cycle can be observed in aging as muscle loss impairs physical capacity and immobility reduces muscle mass [12]. Furthermore, testosterone can also stimulate myoblasts and satellite cells [46]; and IGF-1 stimulates satellite cell proliferation [47, 48] and inhibits protein degradation [49, 50]. All these mechanisms which act in myocyte repair and muscle mass preservation are blunted in aging.

The role of increased protein catabolism on muscle changes is less understood [51]. The main proteolytic pathways can be found in skeletal muscle: lysosomal, Ca^2+^ dependent, caspase dependent, and ubiquitin-proteasome dependent pathways [52]. The ubiquitin-proteasome system is one of the most important pathways responsible for intracellular degradation of striated muscle proteins [53–55]. However, its role remains controversial during aging; recent data suggests that protein degradation is more likely mediated by the Ca^2+^ dependent calpain and autophagy pathways than the ubiquitin-proteasome system [50, 56]. Under physiological conditions, autophagic processes are closely regulated; autophagy inhibition leads to intracellular garbage accumulation, while its excessive activation is associated with autophagic cell death and loss of muscle mass [57]. The PI-3K/Akt/mTOR pathway, as well as stimulating protein synthesis, inhibits protein degradation [4]. PI-3K/Akt inhibits forkhead box transcription factor O (Fox-O), a potent inductor of the ubiquitin-proteasome system, and mTOR decreases caspases activity. Furthermore, physical inactivity stimulates Fox-O, which can also inhibit the mTOR pathway [4]. Also the reduction in number and activity of lower motor neurons contribute to inactivity and muscle breakdown [29].

Satellite cells are a major source of muscle regeneration; however, it is unclear whether a decrease in their number or regenerative capacity is involved in aging muscle changes [50, 58]. Another pathway which may be involved in muscle atrophy is myostatin, a member of the transforming growth factor-β family. Myostatin is secreted by cardiac and skeletal muscle cells and acts locally by negatively modulating skeletal muscle mass. Myostatin inhibits the Akt/mTOR pathway, activates Fox-O, and decreases satellite cells number and regeneration [45, 50]. Despite all these effects, the role of myostatin on aging and different muscle wasting conditions is not completely clear [4, 59–61].

Mitochondria integrate several cell signals including energy supply, ROS generation, and apoptosis. A decrease in mitochondrial content and function was observed during ageing and may contribute to reduced mitochondrial bioenergetics and increased mitochondrial-derived ROS production and apoptotic cell death [56, 62–65]. Apoptosis decreases muscle size by reducing fiber number and decreasing the nuclear-to-cytoplasm ratio by targeted myonuclei removal [29]. Mitophagy, the removal and degradation of dysfunctional portions of mitochondria, is also changed during aging [56, 66]. Peroxisome proliferator-activated receptor gamma coactivator 1-alpha (PGC-1α) is a key regulator of mitochondrial biogenesis in skeletal muscle. Recent data suggested that a reduction in PGC-1α signaling is associated with decreased Akt and mTOR expression in ageing [67]. Furthermore, overexpression of PGC-1α in aged mice attenuated mitochondrial impairment, apoptosis, autophagy, proteasome activity, and muscle loss [67]. Mitochondrial changes are therefore considered to greatly contribute to age-associated muscle alterations [29, 50, 56, 68].

More recently, the role of iron on muscle changes has attracted great interest. Iron deficiency has been linked to several alterations such as decreased physical capacity and muscle mass; altered oxidative-to-glycolytic fiber ratio; reduced myoglobin pool; decreased mitochondria and mitochondrial cristae density; and reduced oxidative metabolism with increased glycolytic activity [69]. Despite this information suggesting iron deficiency plays a role in impairing physical capacity, iron status has been poorly addressed in age-associated muscle changes.

Finally, inflammation is not considered an important factor causing muscle loss in healthy ageing [15]. Although increased interleukin-6 levels can occur in advancing age [67], and elevated tumor necrosis factor alpha (TNF-α) in elderly individuals has been associated with reduced muscle mass and strength [6], it is not clear whether inflammatory activation is due to ageing alone or underlying comorbidities. Furthermore, inflammatory pathways involving NF-κB are typically not activated in sarcopenia [4].

## REACTIVE OXYGEN SPECIES AND SKELETAL MUSCLE AGEING

Oxidative stress is characterized by increased levels of ROS and/or reactive nitrogen species. It can be caused by decreased antioxidant capacity due to low concentrations of antioxidants and impaired antioxidant enzymes activity, and/or by increased ROS production [70]. At physiological concentrations, ROS play essential roles in redox signaling and cell survival by activating or inhibiting enzymes such as mitogen-activated protein kinase (MAPK), phosphatases, and gene-dependent cascades [30]. However, high ROS levels induce alterations or damage to DNA, proteins, and lipids, and can stimulate apoptotic cell death [50, 71].

Skeletal muscles consume large quantities of oxygen and can generate a great amount of ROS. ROS are mainly generated in mitochondria during normal respiration as a by-product of oxidative phosphorylation [29, 71]. They can also be produced in cytosol and membranes in response to different stimuli including growth factors and inflammatory cytokines [71–73]. Several enzymes participate in ROS generation, including xanthine oxidase, nicotinamide adenine dinucleotide phosphate (NADPH) oxidase, and nitric oxide synthase [74]. Skeletal muscle also generates reactive nitrogen species (RNS) [75]. The ROS/RNS-induced modifications include nitration, nitrosylation, carbonylation, and glycation [30]. Under physiological conditions, oxidative stress is neutralized by the antioxidant system, which includes endogenous and exogenous molecules. The main enzymatic defenses are superoxide dismutase, catalase, and glutathione peroxidase [71]. These enzymes can be modified by exercise, nutrition, and aging [76]. Exogenous antioxidants mainly include vitamins (e.g., vitamin E, vitamin C) and minerals (zinc, copper, iron) [30]. The antioxidants maintain muscle redox status and attenuate ROS-induced intracellular changes [30].

Due to the increased oxidative stress levels observed in aged muscle, ROS accumulation has been suggested as playing a role in muscle changes and sarcopenia. The first free-radical theory of aging was proposed by Harman in 1956 [77], who hypothesized that endogenously generated reactive oxidants cause cumulative oxidative damage to macromolecules resulting in the aging phenotype. In aging, oxidant production from several sources is increased, antioxidant enzymes are decreased, and the adaptive response to oxidative stress is reduced. Increases in ROS and RNS production are mainly due to mitochondrial dysfunction caused by age-related mitochondrial DNA mutations, deletions, and damage. Extensive damage to mitochondrial DNA and impaired DNA repair mechanisms in skeletal muscle have been observed with advancing age [29, 78, 79]. Furthermore, the impaired ability of muscle cells to remove dysfunctional mitochondria can contribute to enhanced ROS production [56]. These increased ROS levels cause progressive damage to mitochondrial DNA, thus creating a vicious cycle [29, 80, 81].

As previously reported, faster age-induced decline in Type II fibers can at least be partially attributable to a greater oxidative injury and apoptosis, as this type of fiber has lower mitochondrial content and is more susceptible to atrophy than the Type I fibers with a high mitochondrial content [82, 83]. Increased ROS production also activates the ubiquitin-proteasome system and muscle proteases (i.e., caspases, calpains) leading to protein breakdown [30]. Furthermore, repair systems, such as the proteasomal degradation of damaged proteins, are impaired in aging [30, 84].

ROS and RNS accumulation is also associated with intracellular functional changes in fiber activation at the neuromuscular junction, excitation-contraction coupling, and at cross-bridge cycling within the myofibrillar apparatus [30]. Intrinsic changes in the excitation-contraction process can explain the fact that strength deficit can be more rapid than the concomitant reduction in muscle size in elderly individuals [30].

## PREVENTION AND TREATMENT OF AGEING-ASSOCIATED SKELETAL MUSCLE CHANGES

Despite extensive studies on the molecular pathways involved in age-associated skeletal muscle changes, it has been difficult to develop specific therapies for their prevention and treatment. Different options have been described, mostly evaluated in experimental settings or small clinical trials. Currently, nonpharmacological therapies such as physical exercise and nutritional support are considered the basis for prevention and treatment of age-associated muscle abnormalities [9].

## PHYSICAL EXERCISE

Exercise is the most effective intervention in preventing and treating skeletal muscle changes and sarcopenia in older individuals. Exercise training not only attenuates muscle loss but increases muscle mass and strength, and improves functional capacity and survival [15, 57, 85–89]. However, the molecular mechanisms and signaling pathways involved in exercise benefits are not completely clear.

Anabolic and catabolic muscle pathways are strongly influenced by physical exercise. Regular training improves muscle mass and strength by increasing protein synthesis, number of myofibrils, and fiber cross-sectional area [90]. Exercise increases IGF-1 levels with the subsequent activation of mTOR to induce protein synthesis; mTOR may also be activated by muscle mechanical loading [50, 85]. Furthermore, exercise increases myofibrillar protein through satellite cell activation, and decreases muscle fat infiltration [85]. Besides stimulating muscular anabolism, exercise inhibits protein degradation, an effect probably mediated by the lower levels of oxidative stress following training (see below) [50]. The intensity of autophagic modulation by exercise depends on fiber type and training duration and intensity [57]. Other mediators of muscle loss that exercise may target in ageing are myostatin and Fox-O, which are reduced by aerobic training [91]. Reduced myostatin signaling represses atrogene transcription and consequently protein degradation [50].

Physical training also modulates other muscle cell organelles [92]. Mitochondria are strongly influenced by exercise, which prevents a decrease in their content and function during aging [56, 62]. In fact, the level of physical activity is one of the most important determinants of mitochondrial function in aging muscle [56].

In 1982 Davies et al. [93] observed increased muscle ROS generation in exercised rats. It was later demonstrated that a single bout of exercise exceeding a certain intensity or duration increases ROS production from the mitochondrial respiratory chain or other oxidases and leads to oxidative damage to lipids, proteins, and DNA [94]. However, regular exercise increases ROS formation to a level that may cause significant but tolerable damage, which in turn, can induce beneficial adaptations by up-regulating cellular antioxidant systems and stimulating oxidative damage repair systems [95–100]. In fact, a clinical trial showed that the effects of exercise mediated by a transient increase in ROS production leading to enhanced insulin sensitivity were prevented by antioxidant supplementation with vitamin C or vitamin E [101]. These results suggest that acute ROS production in healthy individuals is required for skeletal muscle adaption to exercise [75]. Thus, contrary to what occurs after acute bouts of exercise, chronic exercise is associated with decreased levels of oxidative stress markers and increased enzymatic and non-enzymatic antioxidant capacity in young, middle-aged, and elderly individuals [29, 102–106]. Muscle biopsies showed reduced oxidative stress and increased catalase expression in lifelong trained older adults compared with their untrained counterparts [50, 107]. Increases in antioxidant enzymes have been associated with improvements in ageing skeletal muscle changes [108]. For example, overexpression of anti-oxidant enzyme Cu^2+^,Zn^2+^-superoxide dismutase in mice prevented age-related muscle impairment [108].

PGC-1α is a central regulator of exercise-induced mitochondrial adaptations and metabolic reprogramming. After exercise, its expression is activated by kinases including MAPK and adenosine monophosphate-activated protein kinase (AMPK), which are stimulated by ROS [29]. It was recently observed that PGC-1α level was enhanced following low intensity, long duration acute swimming and was associated with reduced apoptosis in mice skeletal muscle [109]. Furthermore, increased PGC-1α expression in ageing mice was associated with lower oxidative stress, inflammation, apoptosis, autophagy, and proteasome activation; higher mitochondrial biogenesis; and prolonged survival [67]. Exercise also increases PGC-1α4 isoform, which induces protein synthesis via the IGF-1 pathway and represses myostatin [50].

Improved adenosine triphosphate (ATP) synthesis, oxidative phosphorylation, and Ca^2+^ homeostasis were also observed in elderly skeletal muscles after training [29, 57]. Resistance exercise appears to decrease TNF-α expression in aged skeletal muscle, which may attenuate age-associated muscle changes [110]. Finally, exercise induced favorable skeletal muscle angiogenesis and improved endothelial function in elderly individuals [111, 112].

Despite all the information on exercise modulating signaling pathways, there is little knowledge on the best type, intensity, and frequency of exercise to prevent or treat age-induced muscle loss [113]. Furthermore, not only changes in limb musculature but changes in inspiratory muscles might account for lower exercise capacity in the elderly [12]. Exercise intensity also influences muscle changes. Decreased basal hydrogen peroxide (H_2_O_2_) production in muscle tissue was observed after 16 weeks training at 65% of maximal oxygen uptake (VO_2 max_) [114] and an increase in muscle antioxidant defense was found after 8 weeks of endurance training in older individuals [115]. Although recent publications have suggested that regular high intensity physical activity can be better than moderate intensity in healthy and unhealthy older individuals [116–118], greater physical fitness observed at high intensity exercise increased lipid peroxidation damage more than at a low physical fitness level [117]. Currently, data from literature suggest that optimal aerobic training for improving oxidative/antioxidant balance can be achieved with intensities between the two ventilatory thresholds (50-80% of VO_2_ max) at a frequency of 2-3 sessions per week [71].

Concerning resistance training, it was recently observed that a 12-week resistance training with a frequency of 2 days per week improved muscular strength and oxidative stress in older women and 12 weeks detraining did not completely reverse the changes [119]. Researchers recommend that training protocols should contain sufficient volume for each muscle group (3-5 sets, 10 repetitions) with intensities between 50 and 80% of one repetition maximum [71]. Although current data support the use of both endurance and resistance training in older adults with respect to superior beneficial mitochondrial adaptations and functional outcomes than isolated endurance training, more research is needed to confirm this [29].

## NUTRITION

Nutritional status should be carefully evaluated to identify and prevent protein and micronutrient deficits. All the factors important for muscle function should be addressed. Vitamin D deficiency is common and affects all ages and both sexes. As evidence suggests that vitamin D is important for muscle function, maintaining adequate vitamin D status may be considered in preventing and treating age-associated muscle changes. Thirteen weeks of oral vitamin D supplementation and leucine-enriched whey protein improved muscle mass and lower-extremity function in older sarcopenic individuals [120]. Currently, prospective studies are underway to better define the role of vitamin D on skeletal muscles [121, 122].

As previously reported, iron participates in skeletal muscle function [69]. Although this issue has been poorly addressed in age-associated muscle changes, research on diseases such as heart failure and chronic obstructive pulmonary disease have suggested that iron deficiency may impair physical capacity. Therefore, iron status should be evaluated when muscle dysfunction is present without an apparent reason and iron deficiency treatment should be viewed as an emerging therapeutic target [69].

Ingestion of an adequate amount of high-quality protein in combination with physical activity appears a promising strategy to prevent or treat sarcopenia [123]. However, results of interventions are inconsistent and it is unknown whether specific nutritional therapy alone can reverse muscle loss [12]. Therefore, randomized studies analyzing the effects of nutrition interventions are needed to establish specific recommendations on nutritional support [124]. Guidelines for the nutrition and nutritional support of elderly individuals have been published [125, 126] and will not be discussed here.

## PERSPECTIVES FOR FUTURE

Potential contenders for preventing or treating age-induced skeletal muscle changes have been described. Candidate drugs include myostatin antagonists, follistatin, activin receptor antagonists, ghrelin agonists, selective androgen receptor molecules, megestrol acetate, beta antagonists, espindolol, formoterol, angiotensin converting enzyme inhibitors, and fast skeletal muscle troponin activators [12, 18, 127].

The value of testosterone replacement therapy for older men is currently under intense debate. Ageing is accompanied by reduced protein synthesis and transporter. It was recently shown that these changes may be reversed by dihydrotestosterone treatment [128]. Other studies have also suggested that testosterone increases muscle mass and power [127, 129]. Growth hormone, a modulator of muscle growth and differentiation, has been evaluated to preserve skeletal muscle mass and myocardial metabolism under different conditions [130, 131]. Growth hormone administration to healthy older men increased lean body mass without changing muscle functional parameters [132]. However, due to testosterone and growth hormone potentially limiting side effects [124, 125, 132], research is needed before recommending hormonal supplementation in clinical practice.

Anti-myostatin antibodies have been extensively investigated under different clinical conditions associated with muscle loss. Treatment of elderly mice with an anti-myostatin antibody (ATA 842) for 4 weeks increased muscle mass and strength and improved insulin-stimulated muscle glucose uptake [133]. In a clinical setting, a multicenter study showed that administration of myostatin antibody LY2495655 to subjects 75 years or older who had fallen in the past year increased lean mass and might improve functional muscle power [134]. Additional studies are needed to confirm these results.

Recently, myocardial metabolic modulator trimetazidine (TMZ) was evaluated in skeletal muscle. Trimetazidine prevented hypotrophy of skeletal muscle cells in culture with different hypotrophic agents [135] and improved skeletal muscle strength of elderly mice [136]. Intraperitoneal administration of the newly developed mitochondria-targeted ROS and electron scavenger, XJB-5-131, reversed age-related mitochondrial function alterations and improved contractile properties in skeletal muscle [137]. Oxandrolone improved body composition adaptations to 12 weeks exercise in older women without however increasing muscle function or functional performance beyond that of exercise alone [138]. The benefits of low level laser therapy as an intervention to enhance muscle performance in the elderly is under investigation [139]. Collagen peptide supplementation combined with resistance training increased fat free mass and muscle strength in elderly sarcopenic men compared with training alone [140]. Reduction in histone deacetylase activity had a protective effect in models of neurogenic muscle atrophy [141]. As sarcopenia is associated with a reduction in motor neuron innervation, the potential for histone deacetylase inhibitor butyrate to modulate age-related muscle loss was investigated in older mice. Butyrate treatment starting at 16 months of age showed promising results by attenuating muscle atrophy [142].

Finally, it should be emphasized that a comprehensive geriatric assessment involving a multidisciplinary team enables medical staff to optimize the treatment of older individuals with skeletal muscle changes [143].

In summary, skeletal muscle changes are prevalent in the geriatric population and are associated with impaired outcomes. The main alteration is sarcopenia, which is characterized by structural, biochemical, molecular and functional changes. Muscle loss is a multifactorial condition with an imbalance in anabolic and catabolic intracellular pathways. Oxidative stress seems to play an important role in age-induced skeletal muscle abnormalities. Currently, despite the development of new agents, nonpharmacological therapies such as physical exercise and nutritional support are considered the basis for prevention and treatment of age-associated muscle abnormalities.

## Abbreviations

Akt: serine threonine kinase
AMPK: adenosine monophosphate-activated protein kinase
ATP: adenosine triphosphate
Fox-O: inhibits forkhead box transcription factor O
H_2_O_2_: hydrogen peroxide
IGF-1: insulin growth factor 1
MAPK: mitogen-activated protein kinase
mTOR: mammalian target of rapamycin
NADPH: nicotinamide adenine dinucleotide phosphate
NF-κB: nuclear factor kappa B
PGC-1α: peroxisome proliferator-activated receptor gamma coactivator 1-alpha
PI3K: phosphatidylinositol 3-kinase
RNS: reactive nitrogen species
ROS: reactive oxygen species
TMZ: trimetazidine
TNF-α: tumor necrosis factor alpha
VO_2 max_: maximal oxygen uptake
